# The impact of affective and cognitive empathy on stress in medical students

**DOI:** 10.1186/s41155-024-00336-9

**Published:** 2025-03-11

**Authors:** Madson Alan Maximiano-Barreto, Julia Leles Bueno, Mariana Leles Bueno, Victor Oliveira Wercelens, Julia Guimarães Mauad Ydy, Roberta Perfeito Abrahim, Jed Montayre, Késia Maria Maximiano de Melo

**Affiliations:** 1 Department of Neuroscience and Behavioral Sciences, Faculdade de Medicina de , Av. Tenente Catão Roxo, Ribeirão Preto, SP 2650 Brasil; 2 Medical Students, IMEPAC University Center, Araguari, MG Brazil; 3 School of Nursing, The Hong Kong Polytechnic University, Hong Kong, China; 4 Occupational Therapy Department, Federal University of Santa Maria, Santa Maria RS, Brazil

**Keywords:** Empathy; Medical students, Psychological stresses, Social ability

## Abstract

**Objective:**

To analyze the relationship between empathy and its domains (i.e., affective and cognitive) and stress in medical students.

**Methods:**

An online cross-sectional study with 543 medical students as respondents divided in three groups: pre-clinical cycle (*n* = 173), clinical cycle (*n* = 197), and internship (*n* = 173). The participants completed the sociodemographic questionnaire, the Interpersonal Reactivity Index, and the Perceived stress scale.

**Results:**

The participants were mostly female (71.5%) with an average age of 23.54 (± 4.54) years old. Medical students in the clinical cycle, when compared to the ones in their pre-clinical cycle and internship, showed higher stress levels. Pre-clinical students presented higher levels of total empathy and its affective domain. The highest levels of empathy, especially affective empathy, were associated with higher stress levels in all three groups. Regarding cognitive empathy, there was no observed association with stress among the groups.

**Conclusions:**

The levels of empathy and its affective domain correlated significantly with the stress indicators among students of different cycles (i.e., pre-clinical, clinical cycle, and internship).

## Introduction

Empathy is an important ability for health care providers (e.g., physicians) (Peisachovich et al., 2023). Empathic physicians communicate better with patients (Wang et al., 2022; Yu et al., 2022), offering greater satisfaction (Yu et al., 2022) to patients with regards to treatment decision-making (Martikainen et al., 2022). However, studies have shown that higher levels of empathy are correlated with higher levels of psychological concerns (e.g., stress) among care providers (Maximiano-Barreto, Fabrício et al., 2022), and thus may have implications for the provision of care.

Empathy is widely studied in the diverse fields of health care, especially medicine (Akgün et al., 2020; Maximiano-Barreto et al., 2020; Santo et al., 2023; Wercelens et al., 2023). This multidimensional construct has several conceptual definitions (Davis, 1980, 1983; Decety & Jackson, 2004; Mercer & Reynolds, 2002). As defined by Davis (1980), empathy refers to a human ability characterized by two main domains: cognitive and affective empathy. With the affective component, one positions oneself in another person’s shoes, feeling the same emotions. With the cognitive component, one understands the emotions of the other person but is able to separate these emotions (Davis, 1980, 1983).

Empathy strengthens the doctor-patient relationship (Halpern, 2003; Neumann et al., 2009). Higher levels of empathy may assist health care providers in better understanding the needs of patients, increasing their professionalism as physicians. In several studies, patients who received care from health providers with higher empathy levels had better clinical outcomes (e.g., better adherence to treatment and a better prognosis) (Arora et al., 2010; Moudatsou et al., 2020; Neumann et al., 2011). However, higher empathy levels – especially in the affective domain – have been associated with psychological concerns (e.g., stress); in contrast cognitive empathy has been inversely associated with such concerns (Decety, 2020; Maximiano-Barreto, Fabrício et al., 2022).

Common factors contribute to higher empathy levels, such as being a health care provider (Santo et al., 2023), being a woman, being older, having been in the course for a longer period of time, etc. (Maximiano-Barreto et al., 2020). These factors have been associated with higher empathy levels among students and professionals in the health field and are also identified in individuals who are more prone to stress (e.g., medical students) (Mayer et al., 2016; Perissotto et al., 2021). Some studies have pointed out that an excessive workload, academic pressure, personal relationships, aspects related to future expectations, financial issues, time management, and balance between work and personal life also contribute to higher stress levels (Lane et al., 2020; Mao et al., 2019; Mayer et al., 2016; Pacheco et al., 2017; Quek et al., 2019).

Stress has different physiological, psychological, and behavioral consequences that are mediated by one’s perceptions (Chang, 2002). In the present study, both psychological and behavioral levels were assessed using the Perceived Stress Scale (Luft et al., 2007). Studies involving medical students have investigated the shift from the pre-clinical to clinical phase (Morrison & Moffat, 2001; Radcliffe & Lester, 2003) and the effect of this transition on the stress level of students (Prince et al., 2005). Professional interactions, an exhaustive workload, contact with patients, and the acquisition of greater knowledge and skills are the main differences between these phases (Godefrooij et al., 2010; Zarshenas et al., 2014) and the need to adapt to new routines (Hill et al., 2018) may contribute to stress.

The mental health and wellbeing of medical students is an important aspect in the educational process, affecting academic performance, the ability to balance studies and a social life, and the ability to demonstrate empathy (Pacheco et al., 2017; Shanafelt et al., 2005). A study conducted with medical students found that higher levels of empathy were associated with higher levels of stress (Worly et al., 2019). The same result was reported in other studies conducted in different contexts (Park et al., 2015; Paro et al., 2014). However, few studies have addressed the relationship between empathy and stress in medical students comparing the pre-clinical, clinical, and internship phases.

Empathy is both a predictive and protective factor with regards to psychological concerns among health care providers (Maximiano-Barreto, Fabrício et al., 2022). Identifying the relationship between empathy (and its affective and cognitive domains) and stress among medical students can enable the development of coping strategies (e.g., empathy training) focused on the cognitive domain, which has the potential to minimize psychological harm among health care providers (Maximiano-Barreto et al., 2024b) without losing this important ability for future relationships and interactions with patients. The years of medical education are associated with higher levels of empathy (Maximiano-Barreto et al., 2020) and stress levels increase throughout the course (Abdulghani et al., 2011). Therefore, the aim of the present study was to investigate the relationship between empathy (and its affective and cognitive domains) and stress in medical students during the pre-clinical, clinical, and internship phases. It is therefore important to assess and compare different stages of the medical course (i.e., pre-clinical, clinical, and internship phases). We expect to find a direct relationship between affective empathy and stress in all three groups (i.e., students in the pre-clinical, clinical, and internship phases). In the opposite direction, that there is cognitive empathy inversely associates with stress indicators between the three groups.

## Methods

### Design, participants, and ethical aspects

A cross-sectional quantitative study was conducted at a private medical school in the municipality of Araguari, state of Minas Gerais, Brazil. Medicine is a six-year course in Brazil, during the last two of which students are required to be interns (e.g., practical internship in different fields of medicine, such as pediatrics, surgery, and clinical medicine) (Wercelens et al., 2023). In addition to the students of the internship, the pre-clinical and clinical medical students also make up this sample. A total of 1200 students were enrolled at the time of study. All students were invited to participate in the survey, however, 543 medical students participated in this study and were divided into three groups: pre-clinical (1st and 2nd years), clinical (3rd and 4th years) and internship (5th and 6th years). Students enrolled from the first to the last year of the course in the year 2021–2022 were included. Students younger than 18 years of age were excluded. This study received approval from the Human Research Ethics Committee ***DE-IDENTIFIED***. Prior to completing the survey, all students agreed to participate by reading and confirming their understanding of the statement of informed consent.

## Measures

### Interpersonal Reactivity Index

The Interpersonal Reactivity Index (IRI) is a research tool used to assess the level of empathy (Davis, 1980, 1983). This scale is composed of 21 statements with response options on a Likert scale ranging from 1 (“does not describe me very well”) to 5 points (“describes me very well”). The IRI enables the assessment of total empathy as well as the cognitive and affective domains. Fourteen of the 21 items (Items 1, 3, 4, 6, 7, 9, 10, 12, 13, 14, 15, 17, 18, and 20) address affective aspects and seven (Items 2, 5, 8, 11, 16, 19, and 21) address cognitive aspects. The total ranges from 21 to 105, with higher scores denoting higher levels of empathy. The present study used the version translated and adapted to Brazilian Portuguese, which has good internal consistency (Cronbach's *α* = 0.75) (Koller & Camino, 2001).

### Perceived Stress Scale

The Perceived Stress Scale (PSS-14) is a research tool developed to assess the presence of stress (Cohen et al., 1983). This scale is composed of 14 items with response options on a Likert scale ranging from 0 (“never”) to 4 (“always”). Seven items address positive aspects (4, 5, 6, 7, 9, 10, and 13), which are scored inversely, and seven address negative aspects (1, 2, 3, 8, 11, 12, and 14). The total ranges from 0 to 56, with higher scores denoting higher levels of stress. The version translated and adapted to Brazilian Portugues has good internal consistency (Cronbach's *α* = 0.75) (Luft et al., 2007).

### Procedures

Students were invited to participate through a public call via social media (i.e., website, Instagram, and Facebook) of the main author’s university. The study was conducted online between July 2021 and June 2022 via the *Google Forms* platform and obtained a 57% response rate. Students who volunteered to participate answered the researcher-developed survey. The questionnaire included sociodemographic characteristics to identify sex, color/ethnicity, age, family income, marital status, schooling levels, etc., and also included the IRI and PSS-14 tools.

### Statistical analysis

Data analysis was performed with the aid of the Statistical Package for the Social Sciences (version 23.0). Sociodemographic and clinical data were expressed as percentage (%), mean (X̅), and standard deviation (σ) values. The Kolmogorov–Smirnov normality test revealed that the data had nonparametric distribution. Thus, the Kruskal–Wallis H test and chi-squared test (χ^2^) were used for comparisons among groups (students in pre-clinical, clinical, and internship phases) regarding continuous variables (i.e., age, empathy, empathy domains, and stress) and categorical variables (i.e., sex, marital status, schooling levels, religion, and others) and the Mann–Whitney U test was used for comparisons between two groups that presented statistically significant differences on the H test. Spearman’s rho correlation coefficients (ρ) were calculated to analyze correlations between numerical variables (i.e., empathy and its affective and cognitive domains and stress). ANCOVA was conducted adjusting for sex and age, as being female and being older are associated with higher empathy levels (Maximiano-Barreto et al., 2020). The correlation coefficients were interpreted as follows: 0.10 to 0.39 = weak correlation; 0.40 to 0.69 = moderate correlation; 0.70 to 1 = strong correlation (positive or negative) (Dancey & Reidy, 2006). A *p*-value *p* ≤ 0.05 was considered indicative of statistical significance in all analyses.

## Results

**Fig. 1:**
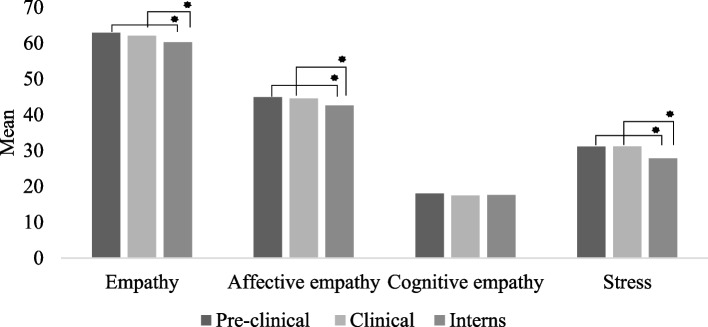
Comparison of levels of empathy, affective and cognitive domains and stress among students in three groupx. *: Difference among groups, Mann–Whitney *U* test

Table 1 displays the sociodemographic and clinical characteristics of the sample. The female sex predominated in the sample (71.5%) and mean age was 23.52 ± 4.64 years. Statistically significant differences among groups were found for the clinical variables: stress (*p* = 0.01), total empathy (*p* = 0.01), and affective empathy (*p* = 0.01). Figure 1 shows the comparisons among groups for clinical variables (i.e., total empathy, affective empathy, cognitive empathy, and stress). Interns had lower levels of total empathy, affective empathy, and stress compared to the students in the pre-clinical and clinical phases. No difference among groups was found regarding the cognitive domain of empathy.

**Table 1:** Sociodemographic and clinical characteristics of medical students

Variables	Total (n = 543)	Pre-clinical phase (n = 173)	Clinical phase (n = 197)	Internship (n = 173)	*H*/χ^2^	*p*
**Sociodemographic variables**		% (n) or X̅ (σ)				
**Age**	23.52 (4.64)	21.76 (4.81)^‡†^	23.46 (4.47)^†¥^	25.35 (3.91)^‡¥^	153.12	**.001**
**Sex**						
Female	71.5 (388)	31.7 (123)	36.1 (140)	32.2 (125)	.08	.96
Male	28.5 (155)	32.3 (50)	36.8 (57)	31.0 (48)		
**Skin color**						
White	79.2 (430)	32.1 (138)	35.8 (154)	32.1 (138)	.19	.91
Non-white	20.8 (113)	31.0 (35)	38.1 (43)	31.0 (35)		
**Marital status**						
Married	5.5 (30)	23.3 (7)	43.3 (13)	33.3 (10)	1.18	.55
Unmarried	94.5 (513)	32.4 (166)	35.9 (184)	31.8 (163)		
**Religion**						
Has religion	80.7 (438)	32.4 (142)	36.5 (160)	31.1 (136)	.73	.69
Has no religion	19.3 (105)	29.5 (31)	35.2 (37)	35.2 (37)		
**Family income**						
Does not know	30.8 (167)	34.1 (57)	37.1 (62)	28.7 (48)	2.58	.86
1 to 4 × MMM*	12.7 (69)	30.4 (21)	34.8 (24)	34.8 (24)		
5 to 8 × MMM	14.0 (76)	32.9 (25)	39.5 (30)	27.6 (21)		
≥ 9 × MMM	42.5 (231)	30.3 (70)	35.1 (80)	34.6 (80)		
**Clinical variables**		X̅ (σ)				
**Stress**	30.07 (10.18)	31.09 (10.60)	31.15 (10.27)	27.82 (9.29)	11.08	**.01**
**Empathy**	61.73 (7.13)	62.90 (6.81)	62.04 (6.75)	60.21 (7.62)	10.86	**.01**
**Affective empathy**	44.06 (7.15)	44.87 (6.65)	44.60 (7.11)	42.62 (7.49)	9.76	**.01**
**Cognitive empathy**	17.67 (3.10)	18.02 (3.14)	17.43 (3.13)	17.59 (3.00)	3.78	.15

^*^monthly minimum wage. Bold type: *p* < .01. ^‡¥^: Difference among groups, Mann–Whitney *U* test. *H*: Comparison among groups using the Kruskal–Wallis *H* test

**Fig. 2:**
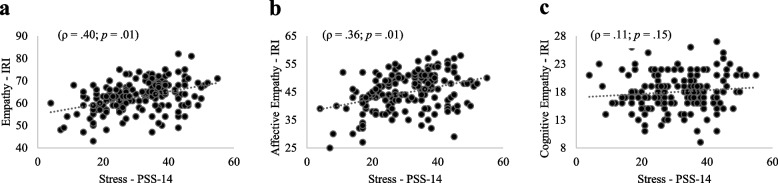
**a**, **b**, **c**. Correlation between stress, empathy, affective domain and cognitive domain among medical students in pre-clinical phase

**Fig. 3:**
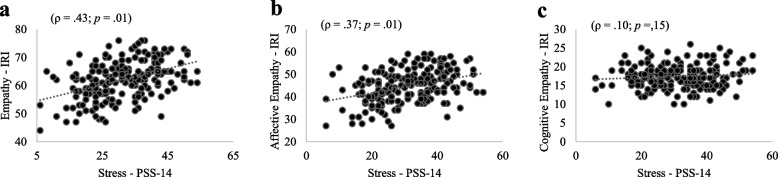
**a**, **b**, **c**. Correlation between stress, empathy, affective domain and cognitive domain among medical students in clinical phase

**Fig. 4:**
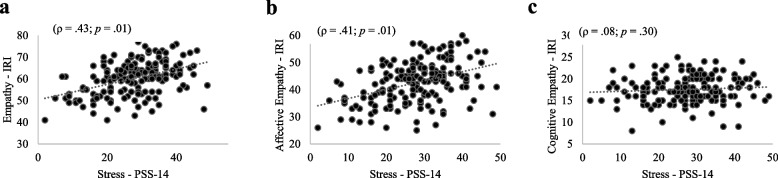
**a**, **b**, **c**. Correlation between stress, empathy, affective domain and cognitive domain among medical students in internship phase

Figures 2, 3, and 4 (a, b, c) show the correlations between stress and total empathy as well as its domains among medical students in the pre-clinical, clinical, and internship phases. Moderate significant and positive correlations were found between stress and total empathy (ρ = 0.40; ρ = 0.43; ρ = 0.43 for pre-clinical, clinical, and internship phases, respectively) as weak significant and positive correlations between stress and affective empathy (ρ = 0.36; ρ = 0.37; ρ = 0.41 for pre-clinical, clinical, and internship phases, respectively). In contrast, no significant correlation was found between stress and cognitive empathy in the three groups. ANCOVA adjusted by sex revealed relationships between stress and total empathy [*F* (1.54) = 85.30;* p* < 0.01] as well as between stress and affective empathy [*F* (1.54) = 60.92; *p* < 0.01] irrespective of sex. Likewise, ANCOVA adjusted by age revealed relationships between stress and total empathy [*F* (1.53) = 11.23; *p* < 0.01] as well as between stress and affective empathy [*F* (1.53) = 6.13; *p* < 0.01] irrespective of age.

## Discussion

The main purpose of the present study was to investigate the relationship between empathy (and its affective and cognitive domains) and stress in students in the pre-clinical, clinical, and internship phases of medical school. We found that both total empathy and its affective domain were directly correlated with higher stress levels. In contrast, no correlation was found between cognitive empathy and stress in any of the three groups. We also found that levels of both empathy and stress decreased as the course progressed. These results are similar to findings described in the literature regarding both total empathy (Akgün et al., 2020) and stress (Abdulghani et al., 2011), with a reduction in the levels of these variables as the course advances.

Total empathy was positively correlated with stress, which is similar to findings described in previous studies conducted with medical students (Park et al., 2015; Paro et al., 2014; Worly et al., 2019). However, one study also conducted with medical students found no association between these variables (Wahjudi et al., 2019). A recent systematic review conducted with care providers of older adults found that higher levels of empathy were directly associated with psychological concerns (Maximiano-Barreto, Fabrício et al., 2022). Besides issues consolidated in the literature with regards to stress (Lane et al., 2020; Mao et al., 2019; Mayer et al., 2016; Pacheco et al., 2017; Quek et al., 2019) and empathy (Maximiano-Barreto et al., 2020), a possible explanation for the association found is the fact that this study was conducted during the period of the COVID-19 pandemic.

The impacts that the COVID-19 pandemic has generated on the education process (e.g., changes from face-to-face activities to online) may justify the higher levels of stress among pre-clinical students. Jhajj et al. (2022) assessed the impact of the COVID-19 pandemic on medical students worldwide. The authors found that this variable (i.e., pandemic) contributed to increased stress in this population. A study conducted with 165 medical students from an Irish university showed that changes in the teaching process (e.g., online activities, online assessments) increase the levels of stress perceived by these students (O'Byrne et al., 2021). Moreover, these changes caused greater social isolation, a factor that corroborates for increased stress (Faeck Jaafar et al., 2022). But still, it is important to evaluate the current moments, since studies show (e.g., Wercelens et al., 2023) that students who endure on the front lines of the COVID-19 pandemic were more impacted when compared to those who do not tolerate on the front lines.

A study conducted with medical students found a 9% increase in empathy levels comparing pre-pandemic and pandemic periods (Awadalla et al., 2022). Therefore, both students who had newly entered the course (i.e., pre-clinical phase) and those who had been in the course for a longer period of time (e.g., clinical and internship phases) had to adapt to the unusual situation and develop new abilities (Aslan & Pekince, 2021). Moreover, social distancing exerted an influence on the development of different social abilities (Freitas et al., 2020), among which is empathic ability. As we found the higher levels of empathy among the students in the pre-clinical phase, the context in which the study was conducted can also be considered a relevant factor. Indeed, one study conducted with students of the health field found higher levels of empathy during the COVID-19 pandemic compared to the previous year (i.e., pre-pandemic period) (Ghaus et al., 2020).

Another possible explanation for the decreasing levels of empathy throughout the course is the reduction in disciplines related to humanization. A review found that humanization and empathy are correlated (Carmo et al., 2020). The early years of health courses (e.g., medicine) have disciplines related to this aspect (i.e., humanization), which enables increased levels of empathy (Santo et al., 2023). However, the clinical and internship phases have more technical disciplines (Gonçalves & Benevides-Pereira, 2009; Serra et al., 2021), which may contribute to this decrease in empathy levels.

Besides total empathy, the affective domain was also correlated with stress in the three groups. As mentioned above, affective empathy enables individuals to put themselves in someone else’s shoes, experiencing the same feelings and emotions (Davis, 1980). We believe that the academic curriculum could contribute to increasing this domain of empathy domain through the practice of humanization (O'Sullivan et al., 2012). Humanization can have a positive impact on the quality of care offered and patient satisfaction, among other consequences (Benevides & Passos, 2005), enabling individuals to exhibit affective empathy. However, this domain of empathy has implications with regards to the mental health of health care providers. A study by Maximiano-Barreto, Fabrício et al. (2022) found that affective empathy is the domain associated with stress.

No correlation was found between cognitive empathy and stress in the three groups. However, systematic reviews with different populations (e.g., caregivers of older people, medical students) reported that the cognitive domain of empathy can be considered a protective factor for stress, depression, anxiety, burnout and burden (Cairns et al., 2024; Maximiano-Barreto et al., 2024a). Another review conducted with medical students reported the same result (Decety, 2020).

From the biological standpoint, studies have identified that the affective domain of empathy and psychological concerns activate the same regions of the brain (Decety et al., 2016; Shamay-Tsoory, 2013). When an individual exhibits affective empathic behavior, the amygdala, hypothalamus, and anterior insula are activated; these regions are also triggered when a person exhibits psychological concerns (e.g., stress) (Aupperle et al., 2012; Balderston et al., 2020). Conversely, Blix et al. (2013) found that the dorsolateral prefrontal cortex has reduced activity when exposed to stressful situations. Thus, the stimulation of the dorsolateral prefrontal cortex reduces reactivity of the amygdala (Ironside et al., 2019), which is activated in stressed individuals and those with higher levels of affective empathy. Therefore, both empathy and stress are associated with physiological and behavioral changes.

Given that empathy is an ability that can be learned, empathy training focused on the cognitive domain needs to be developed with the aim of minimizing negative impacts (e.g., stress) (Maximiano-Barreto, Ottaviani et al., 2024b). Empathy training is a stress-coping strategy (e.g., mindfulness) that has been conducted with students and professionals in the health field (Shiralkar et al., 2013). Such interventions have the potential to maintain and/or enhance empathy levels in medical students, in addition to developing strategies for coping with stressful factors.

Some limitations of this study should be considered, such as the cross-sectional design, which does not enable the analysis of analyze causality; the use of self-administered scales, as students may have different interpretations of the items; the fact that the sample was recruited from a single education institution; and the online data collection, which may have impeded the participation of some students. A limitation regarding internet access may have deterred students from finishing the questionnaire. Furthermore, a higher frequency of female individuals, since as previously demonstrated (see: Maximiano-Barreto et al., 2020) women have higher levels of empathy and stress (Abdulghani et al., 2011; Graves et al., 2021) when compared to men. But it is noteworthy that previous studies (e.g., Maximiano-Barreto et al., 2022; Wercelens et al., 2023) showed that regardless of this variable (i.e., sex), there is a relationship between the variables and before an ANCOVA was performed by adjusting the variables for sex. Finally, both stress and empathy were assessed using self-report instruments, which can produce biases in terms of social desirability and self-perception capacity.

Although we have identified the limitations described above, this study supports future research with medical students in Brazil and elsewhere. It is important to consider studies with different methodological designs (e.g., longitudinal and multicenter) that evaluate the association of these variables with public higher education populations. In addition, studies with probabilistic and homogeneous samples regarding sex are necessary. Empathy training with a focus on cognitive empathy has been developed to decrease the levels of psychological concerns in caregivers of elderly people, as demonstrated in a systematic review (see: Maximiano-Barreto et al., 2024b). Thus, thinking about future interventions thinks about the empathy aspect, it can be more a strategy to minimize stress, for example.

## Conclusion

The present cross-sectional study conducted with Brazilian medical students in the pre-clinical, clinical, and internship phases of the course analyzed the relationship between empathy (and its domains) and stress. Based on the results of this study, we have identified that stress levels are directly correlated with empathy levels in medical students. This correlation was found irrespective of the group analyzed (i.e., students in the pre-clinical, clinical, and internship phases), confirming the hypothesis raised. We found reductions in stress as well as total and affective empathy scores from the pre-clinical phase to internship phase. Despite this reduction, the two variables were correlated. The present findings underscore the importance of conducting interventions in the academic environment in an attempt to minimize negative impacts (e.g., stress) throughout the course while maintaining the empathic ability, which is important to the health professional-patient relationship.

## Data Availability

Due to the nature of this research, the participants did not agree for their data to be shared publicly. Therefore, no supporting data is available.
